# Role of platelet-rich plasma in pelvic floor disorders: A systematic review

**DOI:** 10.18502/ijrm.v21i12.15034

**Published:** 2024-01-25

**Authors:** Eighty Mardiyan Kurniawati, Nur Anisah Rahmawati, Gatut Hardianto, Hari Paraton, Tri Hastono Setyo Hadi

**Affiliations:** ^1^Department of Obstetrics and Gynecology, Faculty of Medicine, Universitas Airlangga, Surabaya, Indonesia.; ^2^Faculty of Public Health, Universitas Airlangga, Surabaya, Indonesia.

**Keywords:** Platelet-rich plasma, Pelvic floor disorders, Women.

## Abstract

**Background:**

Management for pelvic floor disorders needs to be improved. Platelet-rich plasma (PRP) offers an innovative treatment in general medical care to promote cell regeneration.

**Objective:**

This review aims to investigate the role of PRP in pelvic floor disorders.

**Materials and Methods:**

6 international databases were accessed using several keywords namely PubMed, Science Direct, Cochrane Library, ProQuest, Google Scholar, and Scopus. The inclusion criteria were articles written in English, published in 10-yr period from 2012 until 2022, and investigated the relevant topic. This systematic review followed PRISMA guideline.

**Results:**

644 articles were found in several databases and 15 articles met the criteria. Management for pelvic floor disorders needs to be improved, but there are still many challenges, such as less effective treatments, risk of recurrence, and postoperative wound healing. PRP offers an innovative treatment in general medical care to promote cell regeneration. A total of 644 articles from the database were found, but 15 studies met the criteria. A total of 600 women with various pelvic floor disorders treated with PRP were analyzed. PRP positively impacts female sexual dysfunction, perineal trauma, vulvovaginal atrophy, stress urinary incontinence, vesicovaginal fistula, perineal rupture, and pelvic organ prolapse. Dosages, preparation techniques, injection techniques, and additive materials are varied. Most studies do not report side effects from the therapy, but the urinary disorder complaints must be paid attention to.

**Conclusion:**

PRP can be used to manage pelvic floor disorders. Future studies should clarify and standardize the dose in each case and how to make PRP produce the best results.

## 1. Introduction 

Women in developed and developing countries can experience pelvic floor disorders during their reproductive cycle. The risk of developing the disease increases with age. Older women complain of pelvic floor disorders more frequently than younger women, resulting in increased visits to health facilities (1). A cross-sectional study of the female population in the United States found that 23.7% of women may have pelvic floor disorders, and this prevalence doubles in women aged 80 yr (2). Greater discomfort due to symptoms of pelvic floor disorders correlates with poorer quality of life (3). Treatment of pelvic floor dysfunction has evolved for better management (4). Unfortunately, better treatment must be done because the existing treatment is not fully satisfactory. Recurrence and surgery can be repeated (5). In cases of fistula, postoperative wound healing is a challenge (6). Patient outcomes will improve as the quality of service improves (7).

Harmonious relationships among muscles, nerves, connective tissue, and bones establish the structure and function of the pelvic floor. If this relationship is disrupted, women will complain of pelvic floor disorders (8). Musculoskeletal health is related to pelvic floor physiological functions (9). Chronic pelvic pain can appear as a form of disruption of this system (10).

Platelet-rich plasma (PRP) is frequently discussed in literature. PRP is a derivative of whole blood containing a supraphysiological concentration of platelets (11). PRP can help regenerate damaged tissue. The resulting cytokines can serve as an alternative treatment. Previous research used studies with rats as experimental animals. PRP administration impacts tendon healing in the initial phase and produces greater mechanical resistance (8). The inflammatory, regeneration, and remodeling phases are assisted by the presence of PRP (9).

The use of PRP has increased in the last 10 yr. This is because PRP is a part that comes from the human body itself and contains growth substances 3-5 times greater than plasma. In gynecology, the problem of Lichen sclerosis or cervical ectopia is treated with PRP and has shown promise. In addition, PRP is a simple substance, side effects are small and easy to find (12).

This study aims to explore the role of PRP in pelvic floor disorders through a systematic review study, specifically to evaluate the effectiveness and safety of PRP in women with pelvic floor disorders.

## 2. Materials and Methods 

### Study design and search strategy

The preparation of the review follows the PRISMA guidelines. The international databases, namely PubMed, Science Direct, Cochrane Library, ProQuest, Google Scholar, and Scopus were accessed to search for peer-reviewed papers relevant to the topic. Pelvic floor disorders were conditions that affect the proper function of the female pelvic organs.

The search used several relevant keywords that match the medical subject heading terms and word synonyms. Articles were searched using the following keywords: “urogynecology”, “pelvic floor disorders”, “platelet-rich plasma” and their synonyms. In addition, pelvic floor disorders were searched in detail based on the name of the disease. The term pelvic floor disorders in this study covered 6 problems including pelvic organ prolapse (POP), stress urinary incontinence (SUI), female sexual satisfaction, perineal trauma, vulvovaginal atrophy, and vesicovaginal fistula. Table I shows the terms, keywords, and outcomes. Articles not found in the database but found through the bibliography of other articles were also selected in the review. Table I presented the terms, keywords, and outcomes.

**Table 1:** The terms, keywords, and outcomes


**Term **	**Keywords**	**Outcome**

**POP**	“pelvic organ prolapse” OR “uterine prolapse” OR “rectocele” OR “cystocele” OR “female genital prolapse” AND “platelet-rich plasma”	P-QOL
**SUI**	“stress urinary incontinence” OR “urge incontinence” OR “enuresis” AND “platelet-rich plasma”	GRA, VAS, ICIQ-bladder diary, I-QOL questionnaire, UDI-6, cough stress test
**Female sexual satisfaction**	“female sexual satisfaction” OR “female sexual dysfunction” OR “female sexual arousal disorder” AND “platelet-rich plasma”	FSFI score, orgasm subdomain scores, FGSIS genital perception scores, FSDS-R scores, Rosenberg scale scores
**Perineal trauma**	“perineal trauma” OR “laceration” OR “perineal tears” AND “platelet-rich plasma”	Vascularity, pigmentation, the patient's scar, VAS, vaginal tear recovery
**Vulvovaginal atrophy**	“vulvovaginal atrophy” OR “Genitourinary syndrome of menopause” OR “vaginal atrophy” OR “urogenital atrophy” OR “atrophic vaginitis” AND “platelet-rich plasma”	VHI, VAS, FSD score
**Vesicovaginal fistula**	“vesicovaginal fistula” AND “platelet rich plasma”	Neovascularization and remodeling of surrounding tissue, Stabbatsberg self-rating scale
POP: Pelvic organ prolapse, SUI: Stress urinary incontinence, P-QOL: Prolapse quality of life, GRA: Global response assessment, VAS: Visual analog scale, ICIQ: Incontinence questionnaire, I-QOL: Incontinence quality of life, UDI-6: Urogenital distress inventory, FSFI: Female sexual function index, FGSIS: Female genital self-image scale, FSDS-R: Female sexual distress scale-revised, VHI: Vaginal health index, FSD: Female sexual distress		

### Study selection and outcome measure

Articles discussing the use of PRP in cases of urogynecology and pelvic floor disorders were suitable for review. Study selection was done based on the inclusion and exclusion criteria. Articles published in 10-yr-period from 2012-2022, in English and discussed the relevant topics were included in the review. All studies, observational designs such as case reports, case-series, cross-sectional, cohort, experimental designs, and randomized controlled trials, were considered for inclusion in the review. The exclusion criteria in this study were animal studies, letters to editors, review studies, abstracts without full text, articles written in the language of certain countries other than English, and discussed disorders in males. They did not write a complete method of making PRP until the process of giving it to patients. Titles and abstracts are reviewed first to determine the topic's suitability, discussed and then evaluated with inclusion and exclusion criteria. PICO framework in table II.

**Table 2:** PICO framework


**Criteria**	**Determinants**
**P (Population)**	Women with urogynecology and pelvic floor disorders
**I (Intervention)**	Administration of PRP
**C (Comparison)**	Not given PRP or the provision of placebo and/or conventional therapy
**O (Outcome)**	Indicators of successful therapy in various cases, according to table I
PRP: Platelet-rich plasma	

### Quality assessment

The physiotherapy evidence abbreviated PEDro scale is used to assess the quality of articles in research. This critical appraisal tool has a 10 item scale. The internal validity of clinical trials was assessed in 9 items. External validity on 1 item. The maximum score is 10/10 (excluding external validity items). A high quality research design is concluded at a score of 7. A score of 5-6, the study indicates a moderate-quality study design, and a score < 5 indicates a low-quality study design (13). Guidelines from the Joanna Briggs Institute (JBI) 2017 were used to assess the quality of case reports. JBI and collaborators developed this tool and it was approved by the JBI Scientific Committee after extensive peer review. The conclusion from the JBI analysis shows whether the article is included (14). Quality analysis of cohort studies using critical appraisal skills program (CASP). The core CASP checklist has been tested on healthcare practitioners. This randomized controlled trail underwent a systematic review according to the 1994 JAMA user guide. The CASP results were categorized into moderate-low overall quality (15). The quality of article was presented in tables III, IV, and V.

### Data extraction and synthesis

Titles and/or abstracts were selected independently by the authors using Microsoft Excel. Conformity assessment used Cohen's Kappa Consistency Test. If a disagreement occurs, involve a third party. Researchers identified relevant results, extracted them, sorted them and then identified themes and subthemes. The results of the narrative synthesis were carried out by all authors and are presented in table VI. Data collected are 1) author's name, 2) year of publication, 3) country, 4) type of study, 5) sample, 6) type of case, 7) dose and treatment technique, 8) outcome measurements, 9) findings, and 10) effects side effect. The results of statistical analysis in the manuscript were included, such as the p-value. Case reports and series were to be pooled separately for descriptive analysis. In this manuscript, no meta-analysis could be carried out due to the different types of cases, the limited number of articles, and the heterogeneous population.

## 3. Results

### Study selection and study characteristics

**Figure 1:**
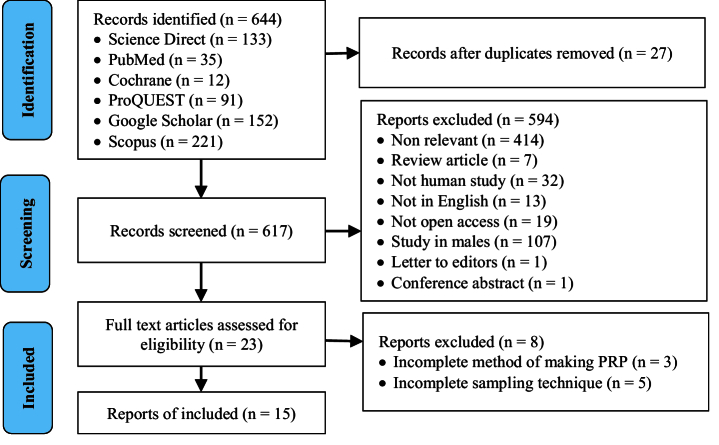
PRISMA flow chart.

644 articles were found in several databases. There were 27 duplicate articles. 594 articles were excluded because they were not relevant to the topics discussed, review articles, not studies on humans, not written in the English language, not open access, male studies, letters to the editor, and abstract conference. Finally, 23 articles were assessed for eligibility, and 15 were found to meet the criteria. The agreement between the 2 independent reviewers on the title/abstract and full text accessed using the KAPPA statistics was 0.747 (99% agreement), respectively. The PRISMA flow chart is shown in figure 1.

### Quality assessment

The quality assessment results of the manuscripts are shown in tables III, IV, and V. The results of the quality of the cohort manuscripts using CASP indicate that the 5 studies are in the moderate category. The results of the PEDro assessment showed that 5 articles were in the medium category, and 1 article was in the good category. The results of the case report assessment showed that the manuscript is worthy of being included in the review.

### Study characteristic 

Study characteristics are shown in table VI. This systematic review involved 15 studies on PRP use in various cases of pelvic floor disorder, including female sexual dysfunction, perineal trauma, vulvovaginal atrophy, SUI, vesicovaginal fistula, perineal rupture, and POP. A total of 600 women were included in this systematic review. The number of study participants was broken down into 164 patients with sexual dysfunction and orgasmic disorder, 1 patient with pain during urination, defecation, coitus, during menstruation, walking and sitting, 20 patients with vulvovaginal atrophy, 179 SUI patients, 16 patients with recurrent vesicovaginal fistula, 210 postpartum patients with perineal rupture, and 10 patients with POP.

### Production technique

The production technique starts by taking blood. 4 studies mention the location in the human body for collecting blood samples. Peripheral blood was collected from superficial saphenous vein in the cubital area of the arm. The centrifugation procedure is known to differ from one study to another. There is a one-spin centrifugation speed, namely 3200 rpm for 8 min, 6000 rpm for 6 min, 1500 rpm for 8 min, 5000 rpm for 5 min, 1500 g for 5 min, and 3400 rpm for 15 min. In addition to one spin, the centrifugation process can be carried out using 2 spin methods, namely, the first spin was at 2500 rpm for 3 min, and the second spin was at 4000 rpm for 15 min, or by first 800 rpm for 10 min, then 15 min at 3500 rpm.

### Injection technique and additives

2 studies added activating ingredients such as calcium chloride. Another 2 studies added hyaluronic acid (HA). PRP-HA injection appears to be a promising method to increase vaginal mucosa trophic and hydration for the treatment of vesicovaginal fistula in postmenopausal breast cancer patients with contraindications to hormone therapy. The combined injection technique of PRP and HA aids in enhancing the modified Stabbatsberg scale and vulva-perineal rejuvenation.

The injection technique depends on the problem at hand. In cases of sexual dysfunction, injections are performed on the anterior vaginal wall and the clitoris. In perineal trauma, it is injected into the perineum. In SUI, it is injected into the urethral meatus, external sphincter, anterior lower one-third of the vagina and periurethral area, or anterior vaginal mucosa. In a vesicovaginal fistula, the injection is done transvaginally. The injection is administered subcutaneously, subvaginally, or via intramucosal; however, the technique is not mentioned. Also, the dose given varies. The injection technique and additives are listed in table VI.

### Outcome

The outcome seen refers to each case. The administration of PRP was associated with an increase in female sexual function index (FSFI) scores, subdomain orgasm scores, and genital perception. The female genital self-image scale (FGSIS) scores (p < 0.001 and p < 0.05) but was not related to the results of the Rosenberg scale. The female sexual distress scale-revised (FSDS-R) score showed a minimal increase in stress score observed at the 4 ${\;}^{\text{th}}$ administration (p < 0.001). In the case of vulvovaginal atrophy, the total vaginal health index (VHI) increased significantly (p < 0.0001) at 6 months. The female sexual distress (FSD) score decreased significantly (p < 0.0001, respectively). In the case of SUI, the mean VAS of SUI scores decreased significantly after treatment (p < 0.001, and p < 0.05). Bladder function variables were significantly improved (p < 0.002). Some studies found that symptoms of SUI at 1 month and 6 months experienced a trend of recovery after being given PRP. The younger group showed a better trend but it did not reach statistical significance (p = 0.07). The results of the incontinence questionnaire (ICIQ) study indicates the effect of this method in the treatment of SUI. The results of the incontinence quality of life (I-QOL) assessment after the treatment observed a significant increase (p < 0.01).

Positive results were also found in case reports and case series research. The data were seen from the patient's responses and complaints after being given PRP therapy. In the case of female sexual satisfaction, improved treatment of secondary sexual dysfunction was seen from the use of PRP for pelvic radiotherapy in a cancer patient. In the case of perineal trauma, PRP impacts ending the patient's complaints of severe pain. In the case of a vesicovaginal fistula, the vaginal wall heals without scarring. In addition, the patient did not complain of difficulty urinating or urinary tract disorders. On the Stabbatsberg scale, an increasing modification was found. In the vulva-perineum, rejuvenation occurs by improving vaginal trophics and rehabilitate normal vaginal caliber.

### Adverse effect

Some studies do not discuss the adverse effects experienced. In cases of SUI, adverse effects can occur in a patient (3.8%) (16, 17). Mild hematuria and painful urination were reported in 10 patients (28.6%) (18).

**Table 3:** Critical appraisal skills program checklist for cohort study


**Author, year (Ref)**	**Did the study address a focused issue?**	**Was the cohort recruited acceptably?**	**Was the exposure accurately measured to minimize bias?**	**Was the outcome accurately measured to minimize bias?**	**Have the authors identified all-important confounding factors?**	**Have they taken account of the confounding factors in the design and/or analysis?**	**Was the follow-up of subjects long complete?**	**Were the follow-up subjects long enough?**	**How precise are the results?**	**Do you believe the result?**	**Can the results be applied to the local population**	**Do the results of this study fit with other available evidence**	**What are the implications of this study to practice?**	**Overall quality assessment**
**Sukgen ** ***et al.*** **, 2019 (19)**	Y	Y	Y	C	N	C	Y	Y	Y	Y	Y	Y	Y	M
**Chiang ** ***et al.*** **, 2022 (16)**	Y	Y	Y	C	N	C	Y	Y	Y	Y	Y	Y	Y	M
**Willison ** ***et al.*** **, 2020 (20)**	Y	Y	Y	C	N	C	Y	Y	Y	Y	Y	Y	Y	M
**Soliman ** ***et al.*** **, 2019 (21)**	Y	Y	N	C	N	C	N	Y	Y	Y	C	Y	Y	M
**Gaber ** ***et al.*** **, 2021 (22)**	Y	Y	N	C	N	N	N	Y	Y	Y	C	Y	Y	M
Y: Yes, C: Cannot tell, N: No, M: Moderate overall quality														

**Table 4:** Physiotherapy evidence databases (PEDro) scale assessment reviewed randomized controlled trials and experimental study

**Author, year (Ref)**	**Eligibility specified (Yes/No)**	**Random allocation (0-1)**	**Concealed allocation (0-1)**	**Homogeneity at baseline (0-1)**	**Subject blinding (0-1)**	**Therapist blinding (0-1)**	**Assessor blinding (0-1)**	**85% follow-up (0-1)**	**Intention to treat analysis (0-1)**	**Between group comparison n (0-1)**	**Point and variability measures (0-1)**	**Total PEDro score (0-10)**
**Hersant **et al**., 2018 **	Yes	0	0	1	0	0	0	1	1	0	1	4
**Daneshpajooh **et al.**, 2021 **	Yes	0	0	1	0	0	0	1	1	1	1	5
**Gorlero **et al.**, 2012 **	Yes	0	0	1	0	0	0	1	1	0	1	4
**Jiang **et al.**, 2021 **	Yes	0	0	1	0	0	0	1	1	1	1	5
**Long **et al.**, 2021 **	Yes	1	1	1	0	0	0	1	1	1	1	7
**Dardeer **et al.**, 2022 **	Yes	0	0	1	0	0	0	1	0	1	1	4

**Table 5:** Quality assessment for case report/case series using the Joanna Briggs Institute Critical Appraisal tools (JBI)


**Author, year (Ref)**	**Were the patient's demographic characteristics clearly described?**	**Was the patient's history clearly described and presented as a timeline?**	**Was the current clinical condition of the patient on the presentation clearly described?**	**Were diagnostic tests or assessment methods and the results clearly described?**	**Was the intervention(s) or treatment procedure(s) clearly described?**	**Was the post-intervention clinical condition clearly described?**	**Were adverse events (harms) or unanticipated events identified and described?**	**Does the case report provide takeaway lessons?**
**Kabakci ** ***et al.*** **, 2021 (17)**	Y	Y	Y	Y	Y	Y	N	Y
**Streit-ciećkiewicz ** ***et al.*** **, 2019 (28)**	N	N	Y	Y	Y	Y	Y	Y
**Aguilar ** ***et al.*** **, 2016 (29)**	Y	Y	Y	Y	Y	Y	N	Y
**Melo 2022 (30)**	Y	Y	Y	Y	Y	Y	N	Y
Y: Yes, N: No								

**Table 6:** Effect of platelet rich plasma (PRP) in urogynecological disorders

**Authors, year (Ref)**	**Country**	**Study type**	**Sample**	**Case**	**Dose and treatment techniques**	**Outcome measurement**	**Finding**	**Adverse effect**
					**Blood collection**	**Centrifugation procedure additives injection procedure**	**Additives injection procedure**	**Procedure**			
**Female sexual satisfaction**
**Sukgen **et al.**, 2019**	Turkey	Retrospective cohort study	52 female patients with sexual dysfunction and orgasmic disorder	Female sexual satisfaction	18 mL of venous blood from the cubital area	Centrifugation: 3200 rpm 8 min Volume: 2-3 mL	Calcium chloride (0.5 mL). The treatment duration: 5 min	Location: anterior vaginal wall around the clitoris volume: 4 cc pili Position: 12, 3, 6, and 9 o'clock	FSFI score, orgasm subdomain scores, FGSIS genital perception scores, FSDS-R scores, Rosenberg scale scores	Positive effects showed in FSFI scores, subdomain orgasm scores, genital perception FGSIS scores, FSDS-R score	No
**Gaber **et al.**, 2021**	Egypt	Retrospective cohort study	20 female individuals with FSD	Female sexual satisfaction	9 cc of blood was obtained from the arm	Centrifugation 1: 2500 rpm for 3 min, Centrifugation 2: 4000 rpm for 15 min	NA	Location: anterior vaginal wall and clitoris	FSFI index	The positive effect showed in FSFI scores	Not discussed in the article
**Dardeer **et al.**, 2022 **	Egypt	Experimental study	45 females took part in the study (16 non-FGM/C and 29 FGM/C)	Female sexual satisfaction	5 cc of PRP was obtained from peripheral blood taken from the arm	Centrifugation: 6000 PMS for 6 min	10% calcium chloride in a 1:10 ratio	Location: anterior vaginal wall (1 session)	FSFI index	A positive effect showed in the total FSFI score	No
**Melo, 2022 **	Mexico	Case report	47-yr-old woman	Female sexual satisfaction	The PRP and the PPP were obtained from the extraction of 20 ml of peripheral blood from the patient	Centrifugation: 1500 rpm for 8 min	NA	Location: in the vaginal introitus and vulva Duration: monthly for 4 months	FSFI index	The positive effect showed in the FSFI score	Not discussed in the article
**Perineal trauma**
**Kabakci **et al.**, 2021 **	Turkey	Case report	A 31-yr-old female patient	Perineal trauma	10 mL of the patient’s blood was drawn from the superficial saphenous vein	Centrifugation: 5000 rpm for 5 min	No	PRP treatment was applied 3 times at 3 wk intervals	Vascularity, pigmentation, the patient’s scar, VAS, pain	Positive effects showed in vascularity, pigmentation, the patient’s scar, VAS, pain	Not discussed in the article
**Soliman **et al.**, 2019 **	Saudi German hospital	Experimental study	210 laboring women aged between 22 and 46 yr old	Perineal rupture	8-9 ml of blood from the patient	Centrifugation: 5 min to separate the PRP from the whole blood	NA	Volume: inject 4.5-5 ml of PRP location: subcutaneous and subvaginal	Vaginal tear recovery	The positive effect showed in vaginal tear recovery	Not discussed in the article
**Vulvovaginal atrophy**
**Hersant **et al.**, 2018**	France	Randomized controlled trials	20 postmenopausal breast cancers survivors with VVA	Vulvovaginal atrophy	Participants' blood	Volume: 2 mL of PRP mixed with 2 mL of HA, Centrifugation: 1500 g for 5 min	2 mL of HA	Intramucosal injections were performed at 0, 1, 3, and 6 months	Evaluation of vulvovaginal mucosa changes using the VHI; secondary endpoint: evaluation of dyspareunia and sexual dysfunction based on the FSD score	The positive effect showed in VHI score, improvement in hydration and vaginal epithelial integrity, and FSD score	No
**SUI**
**Chiang **et al.**, 2022 **	Taiwan	Prospective cohort study	26 women with SUI	SUI	A total of 50 ml of peripheral blood is taken	Centrifugation: (1) 200 × g for 20 min at 20°C (2) 2000 × g for 20 min at 20°C. Volume: 5 ml	No	Location: urethral sphincter injection Volume: 5 ml of PRP Position: 2, 5, 7, 10, and 12 o'clock	The primary endpoint was the post-treatment GRA (scored 0-3) score after 4 PRP treatments. A GRA ≥ 2 was considered a successful result. The secondary endpoints included changes VAS of SUI and urodynamic parameters. The follow-up date was 12 months after the 4th PRP treatment	The positive effect showed in VAS of SUI score and the abdominal leak point pressure	No perioperative adverse events or severe complications occurred, except 1 (3.8%) patient-reported straining to self-limited void
**Jiang **et al.**, 2021**	Taiwan	Experimental study	35 patients with SUI due to urodynamically proven ISD	SUI	a 50-mL whole blood specimen was withdrawn	2 centrifugation	No	Location: external sphincter at 5 sites; duration: 5 mL monthly intervals	The primary endpoint was the change in SUI severity as assessed by a VAS (VAS of SUI)	The positive effect showed in the SUI VAS score	Mild hematuria and pain during micturition were reported by 10 patients (28.6%).
**Willison **et al.**, 2020 **	Australia	Prospective cohort study	62 women with SUI	SUI	The 10 mL blood sample	Not stated in the article	NA	Location: the anterior lower one-third of the vagina and periurethral area	The primary outcome was changes in the participants’ symptoms of SUI. Secondary outcomes were related to general bladder function	The positive effect showed in SUI symptoms and bladder function	No
**Long **et al.**, 2021 **	Taiwan	Experimental	20 women	SUI	2 tubes of the patient's blood were 10 mL each	Centrifugation: 3400 rpm for 15 min	NA	Location: anterior vaginal mucosa around the patient's mid-urethra, duration: monthly treatment was given for 3 consecutive months. Volume: 3 mL	Symptoms severity, sexual function before and after the treatment	The positive effect showed in symptoms but no significant changes in sexual function before and after the treatment	No
**Daneshpajooh **et al.**, 2021 **	Iran	RCT	20 women with SUI	SUI	Venous blood (20 mL) was stored using acid citrate dextrose (ACD) anticoagulant and falcon tubes (15 mL)	Centrifugation: (1) 10 min at 800 rpm. (2) 15 min at 3500 rpm	NA	10 patients received periurethral injections of pure PRP, volume: 3 mL, position: 4 points in the middle of the urethra	ICIQ, I-QOL questionnaire, UDI-6, and cough stress test	Positive effects showed in ICIQ, I-QOL, and UDI-6	Not discussed in the article
**Vesicovaginal fistula**
**Streit-ciećkiewicz** et al.**, 2019 **	Poland	Case series	16 patients with recurrent VVF	Vesicovaginal fistula	Whole blood (150-180 mL) was collected from the patients into sodium citrate tubes (ratio 9:1)	The tubes were centrifuged, resulting in PRP volumes of 4-6 mL	NA	Location: transvaginal in 15 patients. In one patient, the injection was made via cystoscopy Position: 4 to 5 points around the edges of the fistula	Neovascularization and remodeling of surrounding tissue	The positive effect showed in any signs of scarring, redness, or granulosa tissue. Moreover, patients did not complain about urination difficulties or urinary tract disorders	No
**Aguilar **et al.**, 2016 **	France	Case report	It is the case of a 39-yr-old primiparous woman referred to our department for sexual dysfunction	Vesicovaginal fistula	The peripheral blood (4 mL per tube)	Centrifugation: 1500 g for 5 min	HA, and inert cell-selector gel (2 mL of PRP for 2 mL of HA)	NA	Stabbatsberg self-rating scale	The positive effect showed in the modified Stabbatsberg scale and a vulvo-perineal rejuvenation	No
**POP**
**Gorlero, **et al.**, 2012 **	Italy	Prospective cohort study	10 patients with POP	POP	The fibrin concentration	Fibrinogen (17 mg/ml of fibrin)	NA	1 ml of PRF solution can cover an area of 3 to 4 cm	ICS score and P-QoL questionnaire	Positive effects showed in symptoms, sexual activity, and the surgical time	No

PRP: Platelet-rich plasma, FSFI: Female sexual function index, FGSIS: Female genital self-image scale, FSDS-R: Female sexual distress scale-revised, FSD: Female sexual distress, NA: Not available, FGM/C: Female genital mutilation/cutting, PMS: Premenstrual syndrome, GRA: Global response assessment, VAS: Visual analog scale, SUI: Stress urinary incontinence, ISD: Intrinsic sphincter deficiency, POP: Pelvic organ prolapse, ACD: Acid citrate dextrose, ICIQ: Incontinence questionnaire, I-QOL: Incontinence quality of life, UDI-6: Urogenital distress inventory, VVF: Vesicovaginal fistula, HA: Hyaluronic acid, ICS: International continence society, P-QoL: Prolapse quality of life, PPP: platelet pellet plasma, VVA: Vulvar and vaginal atrophy, VHI: Vaginal Health Index, RCT: Randomized clinical trial, PRF: platelet-rich fibrin.

## 4. Discussion

This systematic review has shown that PRP effectively improves the outcome for pelvic floor disorder cases. This is evidenced by significant improvement in outcome measurement in each type of case. This may be because wound healing is influenced by the number of growth factors and the cell parts involved (31). In certain patients, such as when hormone therapy is contraindicated, PRP can be used as an option for treating vaginal atrophy. The results are also favorable when viewed from the parameters and patient satisfaction. Patients who experienced POP and were given PRP showed increased collagen concentration and improved SUI. The use of PRP in cases of fistulas also got reliable results. PRP has great potential in the field of urogynecology, but randomized trials of doses and treatment mechanisms need to be studied (32). PRP regulates tissue reconstruction and is effective in experimental models (33).

In the field of orthopedics and plastic surgery, PRP has long been applied. Due to surgical treatment's limitations, its potential use in uterine prolapse has not been studied (31). Blood collection occurs in the arm area, the cubital area, peripheral blood, or the superficial saphenous vein. In principle, blood drawn depends on the need. As much as 3-5 cc of PRP is produced from as much as 30 cc of venous blood. It also depends on various factors, the device used, the collection technique, and the baseline platelet count (34).

However, there are various methods of preparation and regime of administration/dosages. Certain PRP preparation steps, such as centrifugation, need further study. PRP is prepared by a process known as differential centrifugation. The centrifugation procedure is known to differ from one study to another. Either one spin or 2 spin centrifugation methods can be used. The great variability associated with the methods used in the different stages of PRP processing gives different clinical trial results. Although in this study, most results showed positive treatment outcomes (35). In the manufacturing of PRP, there are 2 centrifugation times, namely the initial and the second. In the early stages, centrifugation separates the red blood cells at a constant speed. In the second stage, centrifugation aims to concentrate the platelets so that part of the final plasma volume becomes suspended. In the initial centrifugation, blood cells will separate into 3 layers, namely the top layer, which consists of many platelets and white blood cells, the buffy coat/thin intermediate layer, which is rich in white blood cells, and many red blood cells in the lower layer (34). Previous studies have found the recommended technique for centrifugation is 100-300 g for 5-10 min for the first round and 400-700 g for 10-17 min for the second cycle. In various dermatological conditions, platelet concentrations of 1-1.5 million platelets/µL are recommended in PRP for treatment (36).

Only a few studies have used activating ingredients in certain cases. This raises the question of its function in case management. There is no standardization when it comes to activating ingredients. More research/evidence will be needed to investigate the role and to determine which ingredient is more beneficial, in terms of dosage, etc. Not all studies add activating ingredients such as CaCl. This study also found a platelet activation technique. The physical form of PRP, the number of growth factors, and the release kinetics are affected by each method of PRP preparation. There is no evidence that ideal concentrations are required (37). In addition, PRP activation is not required when injected into soft tissue (38).

Some studies add HA. In many tissues and fluids, HA (also known as hyaluronan) is found naturally but is more abundant in articular cartilage and synovial fluid. Although easy to find, HA content varies greatly across joints and species (37). HA is involved in angiogenesis, reactive oxygen species, chondrocytes, cancer, lung injury, and immune and skin regulation (39). In this study, HA and PRP were used to support each other in increasing positive outcomes, although the administration technique was different. The technique of administration is subcutaneous, subvaginal, and intramucosal injection. PRP has certain types, one of which is platelet-rich fibrin. This type cannot be injected (34).

Most studies show that there are no side effects. However, 2 studies reported side effects or disorders related to urination. PRP has few side effects because it comes from the patient's blood. In addition, PRP is a relatively inexpensive biological material that is easy to produce. This makes PRP superior to synthetic materials (33).

The use of PRP needs to be uniform to find a pattern of administration for the management of pelvic floor disorders. This study could not be meta-analyzed due to the limited number of studies and variations between cases, different outcome measurement techniques, and varied types of research. The preparation method, sample content, and the proposed application are the guidelines for classifying PRP preparations. Centrifugation speed, centrifugation time, and use of anticoagulants cause variations in preparations. The content will also depend on the levels of platelets, leukocytes, and growth factors (40). Different centrifugation guidelines, and the wide variation in platelet concentrations, make it difficult for clinicians to choose among the available PRP. Nonetheless, PRP is commercially available with the advantages of ready-to-use, sterile but high cost and limited volume (34). 19 articles were not open access, so that can cause bias in concluding. In addition, this research is still limited to several databases.

## 5. Conclusion

The available evidence suggests positive therapeutic effects of PRP for various pelvic floor disorders. However, a clinical trial with a standardized PRP preparation, route of administration, and regime/dosage are required to establish its clinical efficacy. PRP is shown to be safe except for a few urinary symptoms, however, the evidence is limited.

## Conflict of Interest

The authors declare that there is no conflict of interest.
